# Region of birth differences in healthcare navigation and optimisation: the interplay of racial discrimination and socioeconomic position

**DOI:** 10.1186/s12939-022-01709-1

**Published:** 2022-08-10

**Authors:** Ivana Paccoud, James Nazroo, Anja K. Leist

**Affiliations:** 1 University of Luxembourg, Institute for Research on Socio-Economic Inequality (IRSEI), Campus Belval, Maison des Sciences Humaines 11, Porte des Sciences, L-4366 Esch-sur-Alzette, Luxembourg; 2 The University of Manchester, School of Social Sciences / Manchester Institute for Collaborative Research on Ageing, The Cathie Marsh Institute (CMI), Humanities Bridgeford Street, Oxford Road, Manchester, M13 9PL UK

**Keywords:** Racial discrimination, Healthcare inequalities, Economic, social and cultural capital, Bourdieu, Healthcare navigation and optimisation

## Abstract

**Background:**

While a large body of research has documented socioeconomic and migrant inequities in the effective use of healthcare services, the reasons underlying such inequities are yet to be fully understood. This study assesses the interplay between racial discrimination and socioeconomic position, as conceptualised by Bourdieu, and their contributions to healthcare navigation and optimisation.

**Methods:**

Using a cross-sectional survey in Luxembourg we collected data from individuals with wide-ranging migration and socioeconomic profiles. We fitted sequential multiple linear and logistic regressions to investigate the relationships between healthcare service navigation and optimisation with perceived racial discrimination and socioeconomic position measured by economic, cultural and social capital. We also investigated whether the ownership of these capitals moderates the experience of racial discrimination in healthcare settings.

**Results:**

We observed important disparities in healthcare navigation among different migrant communities. These differences were explained by accounting for the experience of racial discrimination. Racial discrimination was also negatively related with the extent of healthcare services optimisation. However, the impact of discrimination on both health service navigation and optimisation was reduced after accounting for social capital. Higher volumes of economic and social capital were associated with better healthcare experience, and with a lower probability of perceived racial discrimination.

**Conclusions:**

Racial discrimination plays a substantial role in accounting for inequality in healthcare service navigation by different migrant groups. This study highlights the need to consider the complex interplay between different forms of economic, cultural and social capital and racial discrimination when examining migrant, and racial/ethnic differences in healthcare. Healthcare inequalities arising from socioeconomic position and racism need to be addressed via multilevel policies and interventions that simultaneously tackle structural, interpersonal, and institutional dimensions of racism.

## Background

In the most economically developed countries in Europe, healthcare systems rely on the principal of equity, which some believe to be achieved through the notion of Universal Health Coverage (UHC). However, it is now widely acknowledged that even within this framework there is persistent inequality in the use of healthcare services across different racial/ethnic and socioeconomic groups [1, 2]. Despite these findings, little is known about how racial/ethnic discrimination and socioeconomic position may jointly contribute to unequal use of healthcare services, with most of the healthcare literature focussing on either socioeconomic inequalities or inequalities in access to healthcare services based on race, ethnicity, or migration status. Although not uniform, most of the evidence suggests that people from more advantaged socioeconomic backgrounds visit preventive, specialised and dental services more often, whereas those from more disadvantaged backgrounds, migrants, as well as racial and ethnic minorities are more likely to visit general practitioners [2–6]. In fact, appropriate access to healthcare for a diverse population requires more than simply providing the service: individuals must also be able to effectively navigate the service in order to optimise its benefits. To date, theoretical and empirical research on inequalities in access to healthcare services has generally focused on issues of healthcare service utilisation and unmet healthcare needs, with little attention paid to the issue of navigation and the optimal use of healthcare services [1, 7, 8].

Navigation of healthcare services is a dynamic process during which individuals move through the healthcare system to find and use services [9]. Healthcare service optimisation is an outcome of a successful navigation process, ensuring access to quality services and the effective use of healthcare systems. Both could be considered as key processes in maximising health outcomes. These processes offer a way to understand the difficulties that may arise when individuals from different socioeconomic and racial/ethnic backgrounds face in finding and negotiating the best possible healthcare.

Given that socioeconomic inequalities are strongly associated with race/ethnicity, some scholars have argued that racial/ethnic differences in health outcomes are mainly a reflection of socioeconomic circumstances [10]. Investigators typically adjust for socioeconomic status, thereby ignoring the causal pathways through which these inequalities are shaped, oftentimes using measurements of SES which are not theoretically grounded [10–13]. Bourdieu’s notions of economic, cultural and social capital thus can provide useful insights into the different mechanisms that reproduce health inequalities. His approach emphasises the lifelong accumulation and interplay of material and non-material resources that individuals possess, and which shape their lifestyle choices and practices. Furthermore, there is a growing literature suggesting that in addition to the socioeconomic determinants contributing to health inequalities, exposure to racism may adversely affect access to healthcare services and health outcomes in general [14–17]. Racism has been defined as an organised social system in which the dominant group disempowers and devalues social groups defined as inferior and it can be based on someone’s race, nationality, ethnicity or other markers of social difference [18]. Racism shapes peoples’ life and health opportunities in three interdependent dimensions: the structural (reflecting disadvantaged access to economic, physical and social resources, including the forms of capital identified by Bourdieu), the interpersonal (accounting for everyday expression and experience of racisms, forms of violence that emphasise the devalued and fundamentally insecure status of those with racialised identities), and the institutional dimension (settings where embedded norms, policies and practices foster racial discrimination in processes and outcomes) [11]. When confronted with racism in healthcare institutions, people may feel disempowered, insecure and stressed, which in turn can hinder their effective use of healthcare services.

This paper investigates how the combination of perceived racial/ethnic discrimination and socioeconomic status affects healthcare service navigation and optimisation. A possible mechanism through which different capitals relate to effective use of healthcare services involves having the necessary skills or dispositions to communicate and understand healthcare information. Indeed, individuals with a higher socioeconomic status are often taken more seriously by health professionals [19]. Likewise, effective social networks can help individuals navigate complex healthcare systems [20]. Capitals, in their different volumes and forms, may not only facilitate effective use of healthcare services but may also act as safeguards in that they provide the necessary resources to manage and cope with discriminatory practices, thereby lessening the impact of racism. Indeed, individuals with wider social support networks have better coping strategies and can mitigate some of the negative effects of discrimination [21]. Although there is a large body of evidence documenting the experience of discrimination among different migrant, and racial/ethnic groups, the extent to which discrimination accounts for differences in the effective use of healthcare services is limited.

Therefore, the present study: (i) investigates differences in healthcare service navigation and optimisation by migrant status; (ii) tests whether perceived racial discrimination can account for these differences; (iii) assesses the relationship between the individual and combined roles of economic, cultural, and social capital on these differences, and (iv) assesses whether the possession of capitals reduces the effect of discrimination. We expect to find inequalities in the navigation and optimisation of healthcare services, explained by self-perceived discrimination and differences at the level of the volume of capitals possessed. We also expect to find that some forms of capitals are more effective at moderating the effect of discrimination. The setting of the study is Luxembourg, a country that guarantees universal access to quality healthcare for all, however with some barriers to healthcare access for low-income individuals [22, 23]. Luxembourg provides an appropriate context to study inequalities in healthcare service navigation linked to migration. Indeed, 48% of the population is foreign born [24], with migrants originating from a variety of regions (both from the Global North and the Global South) and through a variety of routes (refugees, unskilled and highly skilled migrants). However, among other European countries, Luxembourg ranked first in the overall prevalence of discrimination based on ethnic or migrant background [25]. In fact, recently the European Commission decided to open an infringement proceeding against Luxembourg due to its failure to adopt EU laws that combat racism and xenophobia [23, 26].

## Methods

This study draws on a cross-sectional survey developed for a wider study of migrants’ access to healthcare services in Luxembourg. Participants were recruited via health providers and community events in areas with ethnically diverse populations. Questionnaires were distributed to migrant and patients’ associations who serve disadvantaged migrant communities. To reach the expat communities, we distributed the questionnaire through social media. Participants were selected if they were over the age 18 and had resided in Luxembourg for more than a year. The survey was offered in both paper and web-based formats and in four languages: Luxembourgish, French, Portuguese, and English. The questionnaire development was informed by interactions with patient representatives. The University of Luxembourg’s Ethics Review Panel approved the study (ERP 18–037).

### Study variables

We used two outcome measures: the success with which individuals navigate healthcare services and the extent to which the use of healthcare services is optimised. The higher the score, the better individuals navigate or optimise healthcare services. The measurements of these two concepts were informed by the literature and by discussions with the patient representatives as they have not been empirically explored in a quantitative manner. To measure navigation of healthcare services we used Sofaer’s (2009) conceptualisation of navigation taking in consideration various individual, healthcare and patient-provider factors. The measure combines four items (Cronbach’s alpha = 0·78): (i) confidence in the ability to find health providers when needed; (ii) confidence in dealing with administrative procedures (iii) confidence in communicating with health professionals (with all three measured on a scale from ‘Not at all confident’ (=0) to ‘Very confident’ (=5), and (iv) how often healthcare providers gave easy to understand medical information (measured on a 4-point scale from ‘Always’ to ‘Never’). The scores were transformed into z-scores and averaged.

Our measure of the optimisation of healthcare services was informed by the Andersen model of effective access to healthcare services capturing individual satisfaction with healthcare services and improved healthcare status [27]. Thus, we measured optimisation using the sum of two items (Cronbach’s alpha = 0.72). The first is the patient’s level of satisfaction with the quality of healthcare services received in Luxembourg: In general, how satisfied are you with the quality of healthcare services received in Luxembourg? This was measured on a 5-point scale from ‘not at all satisfied’ (=1) to ‘very satisfied’ (=5). The second is the patient’s perception of the extent of health improvement after a health appointment. The question asked: To what extent do you think your medical appointments over the past 12 months have led to an improvement in your health? This aims to capture the outcome of a patient’s appointment, measured on a 5-point scale from ‘No improvement at all’ (=1) to ‘Improved to a very great extent’ (=5).

Based on country of birth, participants were classified into broader migrant categories that consider Luxembourg’s migration context and history:

(i) Those born in Northern and Western Europe or North America (NWE&NA): a group largely consisting of migrant ‘expats’ working in the financial sector and in European Union institutions.
(ii) Those born in Southern Europe: a group consisting largely of migrants linked to work in the country’s historically dominant coal and steel industry.
(iii) Those born in Eastern Europe (including the Balkans): this group includes former Yugoslavians who originally arrived in Luxembourg as refugees in the 1990s, and other professions, including employees in European Union institutions.
(iv) Those born in the Global South, mainly Africa, Asia and Latin America: this includes recently arrived refugees (Syria, Afghanistan, and Eritrea) as well as individuals attracted by Luxembourg’s increasingly globalised economy.

We use individuals born in Luxembourg as a reference group.

The measure of discrimination was developed with the patient representatives and was measured through a single item question that aimed to capture participants’ perceived discrimination in relation to the quality of healthcare services received. Respondents were asked: Have you ever felt that the following negatively impacted on the quality of health service you received? Followed by the main reasons for which participant reported discrimination: race/ethnicity, nationality, or religion. This was then coded as a binary variable (yes = 1 for participants who reported discrimination and no = 0 those participants who did not report any discrimination on those dimensions).

We follow Bourdieu’s conception of capitals to categorise individuals’ socioeconomic position:

*Economic capital* was operationalised using two indicators: household income (ranked in five ordinal levels) and homeownership status, which according to Bourdieu represents the institutionalised form of economic capital, classified in three categories (not an owner, partial homeownership, and full homeownership). Both variables were standardised with a z-score and then averaged to create an index of economic capital.

*Cultural capital* was measured through three indicators to reflect its institutionalised, embodied, and objectified dimensions: parents’ and respondents’ educational backgrounds and the number of books present at home during childhood. These were z-standardised and averaged.

For *social capital*, we used an index that captures the specific nature of social contacts who might facilitate easier healthcare system navigation and optimisation. This is, because for Bourdieu, social capital represents both an individual’s network and the resources that can be used through that network over the short and long term [28]. Therefore, we developed more purposeful questions on whether respondents had someone in their personal network able to recommend a good quality doctor, someone they could ask where and how to access healthcare services and someone able to speed up healthcare appointments, all assessed on a binary scale. The scores on these items were summed, resulting in a range of 0 to 3 points (Cronbach’s alpha = 0.77). Models also included age, gender, partnership status, place of residence, and ability to speak one of Luxembourg’s three official languages.

### Statistical analysis

We fitted sequential multiple regression models to examine the predictors of healthcare service navigation and optimisation, namely, perceived racial discrimination, the different capitals, and whether the ownership of capitals moderates the discrimination burden. In the first step, we compared healthcare service navigation and optimisation for individuals with different regions of birth to individuals born in Luxembourg. In the second step, we tested possible associations between individual experiences of perceived racial discrimination with both outcomes. In the next steps, we assessed the contribution of different forms of capital, both individually and jointly, on navigation and optimisation. In the final step, we modelled interaction terms between the capitals and perceived discrimination, to test whether the devaluation of a migrant’s capital stock could be a possible pathway through which racial discrimination impacts on these outcomes.

## Results

Our cross-sectional dataset consists of 386 individuals, with 67% born outside of Luxembourg, and a median age of 42 (IQR: 31;54). The majority of the sample was female (65%). Table 1 shows the unadjusted prevalence of racial discrimination among different migrant categories. While all migrant categories had a higher prevalence of discrimination than those born in Luxembourg, it was highest for those born in Eastern Europe and in the Global South. Regarding the main capital components, those born in Luxembourg tended to have lower levels of cultural capital but were overrepresented amongst those with higher incomes and amongst homeowners. They also had higher social capital scores. Amongst migrants, Eastern Europeans reported higher cultural capital but lower levels of economic and social capital. Individuals born in the Global South reported similar cultural capital levels to those born in Luxembourg but had lower levels of economic and social capital. Finally, those born in NWE&NA and Southern Europe had higher levels of economic and social capital than those born in Eastern Europe and the Global South but differed in terms of cultural capital.

**Table 1:** Prevalence of (self-reported) perceived racial discrimination in the healthcare setting, and distribution of Bourdieu’s forms of capital across different region of birth categories

	Born in Luxembourg (*n* = 125)	South Europe (*n* = 60)	NW Europe & NA (*n* = 92)	Eastern Europe (*n* = 28)	Global South (*n* = 76)
**Self-reported discrimination (%)**	1·5	11·7	13·1	17·9	18·4
**CULTURAL CAPITAL Respondent’s education (%)**					
Up to lower secondary	13·7	14·3	3·3	3·6	10·3
Upper secondary and college	40·5	33·9	21·1	10·7	38·2
University	45·8	51·8	75·6	85·7	51·5
**Mother’s education (%)**					
Up to lower secondary	58·6	62·9	30·8	11·5	31·3
Upper secondary and college	33·6	24·1	39·7	46·2	41·7
University	7·8	13.0	29·5	42·3	27·0
**Father’s Education (%)**					
Up to lower secondary	50	58·5	28·0	3·9	20·0
Upper secondary and college	41·3	26·4	30·7	46·1	40·0
University	8·7	15·1	41·3	50·0	40·0
**Number of books in childhood (%)**					
Fewer than 25 books	38·2	51·7	10·9	8·0	58·7
More than 25 books	61·8	48·3	89·1	92·0	41·3
**ECONOMIC CAPITAL**					
**Respondent income (Euros) (%)**					
Below 2000	9·7	10·2	12·8	8·3	35·2
2000 - 5000	32·5	42·9	37·2	41·7	40·7
5000 - 8000	25·4	22·5	28·2	20·8	18·5
8000 - 13,000	21·9	20·4	15·3	25·0	1·9
More than 13,000	10·5	4.0	6·5	4·2	3·7
**Homeownership status (%)**					
None	29·5	29·3	41·3	51·9	67·7
Partial	41·1	46·6	33·7	40·7	29·0
Full	29·4	24·1	25·0	7·4	3·3
**SOCIAL CAPITAL, mean (SD)**	1·62(1·74)	1·38 (1·19)	1·38(1·18)	·96 (1·14)	·93(1·05)

*NW* North West, *NA* North America.

Table 2 shows the findings from the models examining the association of discrimination and the different forms of capitals with the ability to navigate healthcare services. There were no differences in healthcare service navigation between those born in Luxembourg and those born in NWE&NA, and those born in Southern Europe. However, there was a significant difference for those born in Eastern Europe and the Global South, who both had lower scores (− 1.11 (95% CI = -2.15, − 0.07) and - 0.87 (95% CI = − 1.71, − 0.03) respectively). After including respondents’ perceived discrimination in model 2, these inequalities were reduced and became insignificant. The subsequent models (models 3–5), assessing the role of the capitals in successful navigation showed that individuals with higher economic and social capital are better able to navigate healthcare services. In addition, after including social capital in model 5, the negative association of perceived discrimination with the ability to navigate healthcare services was reduced (from − 2.04 (95% CI = -2.87, − 1.21) to − 1.62 (95% CI -2.45, − 0.80)). In the final, fully adjusted, model for navigation, both social capital and perceived discrimination had a strong association with the ability to successfully navigate healthcare services and that after including these measures in the model the inequalities faced by those born in Eastern Europe and the Global South were strongly reduced, rendering the coefficients statistically insignificant.

**Table 2:** Regression coefficients for the association of perceived racial discrimination and cultural, economic and social capital on healthcare service navigation

	Model 1	Model 2	Model 3	Model 4	Model 5	Model 6
**Region of birth**						
Ref: Luxembourg						
NW Europe & NA	− 0·53	− 0·33	− 0·34	− 0·34	− 0·17	− 0·13
	[− 1·19,0·14]	[− 0·97,0·31]	[− 1·06,0·38]	[− 1·04,0·37]	[− 0·80,0·46]	[− 0·87,0·61]
South Europe	− 0·09	0·01	−0·18	− 0·22	− 0·03	− 0·33
	[− 0·84,0·67]	[− 0·71,0·72]	[− 0·94,0·57]	[− 1·00,0·57]	[−0·73,0·67]	[− 1·11,0·45]
Eastern Europe & Balkans	**− 1·11**	− 0·86	− 0·88	−0·63	− 0·39	−0·15
	[− 2·15,− 0·07]	[− 1·83,0·10]	[− 1·86,0·11]	[−1·70,0·44]	[− 1·32,0·55]	[− 1·31,1·01]
Global South	**− 0·87**	− 0·61	−0·59	− 0·82	−0·35	-0·60
	[− 1·71,-0·03]	[− 1·45,0·22]	[−1·51,0·34]	[− 1·89,0·25]	[− 1·18,0·48]	[−1·56,0·36]
Ref: no discrimination						
**Discrimination**		**− 2·04**	**− 2·40**	**− 2·17**	**−1·62**	**−1·56**
		[−2·87,-1·21]	[− 3·21,-1·56]	[− 3·08,-1·26]	[− 2·45,− 0·80]	[− 2·40,− 0·71]
**Cultural Capital**			− 0·01			0·01
			[−0·14,0·13]			[−0·12,0·13]
**Economic Capital**				**0·21**		0·06
				[0·03,0·39]		[−0·12,0·24]
**Social Capital**					**0·58**	**0·51**
					[0·37,0·79]	[0·27,0·74]
Constant	0·59	0·64	0·49	−0·31	−0·33	−0·66
	[−0·43,1·47]	[−0·30,1·57]	[− 0·49,1·47]	[−1·89,1·28]	[−1·28,0·62]	[−2·07,0·76]
*R*^2^	0·11	0·18	0·23	0·21	0·26	0·30
95% confidence intervals in brackets						

Adjusted for: age, gender, partnership status, area of residence, and language.

*NW* North West, *NA* North America.

Table 3 presents the findings of the model concerned with the degree of optimisation of healthcare services. Although none of the coefficients for migrant groups were statistically significant, model 2 shows a strong negative relationship between perceived discrimination and healthcare optimisation − 1.53, (95% C = − 2.15, − 0.90), a relationship that persisted after including the socioeconomic variables. Assessing the role of each individual form of capital (models 3–5), we found that adjusting for social capital reduced the negative effect of discrimination, and also had a positive effect on healthcare service optimisation in the fully adjusted model, with a coefficient of 0.55 (95% CI = 0.33–0.76).

**Table 3:** Regression coefficients for the association of perceived racial discrimination and cultural, economic and social capital on healthcare service optimisation.

	Model 1	Model 2	Model 3	Model 4	Model 5	Model 6
**Region of birth**						
ref.: Luxembourg						
NW Europe & NA	−0·03	0·08	0·31	0·11	0·24	0·56
	[−0·59,0·53]	[−0·45,0·62]	[− 0·29,0·92]	[− 0·50,0·73]	[− 0·28,0·75]	[− 0·10,1·23]
South Europe	− 0·06	0·05	0·03	0·05	0·14	0·16
	[−0·75,0·63]	[−0·60,0·70]	[− 0·67,0·74]	[− 0·67,0·76]	[− 0·45,0·73]	[− 0·50,0·83]
Eastern Europe & Balkans	-0·36	-0·19	0·06	0·05	0·15	0·80
	[−1·15,0·44]	[−0·97,0·59]	[−0·86,0·98]	[− 0·78,0·88]	[− 0·60,0·90]	[− 0·14,1·74]
Global South	0·06	0·32	0·53	0·36	0·50	0·57
	[−0·70,0·82]	[−0·46,1·10]	[− 0·50,1·55]	[− 0·61,1·34]	[− 0·23,1·23]	[− 0·45,1·58]
**Self-perceived discrimination**						
ref.: no discrimination						
Discrimination		**−1·53**	**−1·79**	**−1·74**	**− 1·28**	**− 1·40**
		[−2·15,−0·90]	[−2·44,-1·14]	[− 2·49,− 0·99]	[− 1·86,-0·70]	[− 2·05,-0·75]
**Cultural Capital**			-0·07			-0·06
			[−0·18,0·04]			[−0·18,0·05]
**Economic Capital**				0·08		0·03
				[−0·05,0·22]		[− 0·12,0·17]
**Social Capital**					**0·48**	**0·55**
					[0·30,0·65]	[0·33,0·76]
Constant	7·51	7·60	7·58	7·59	6·84	6·92
*R*^2^	0·08	0·15	0·16	0·17	0·23	0·28
95% confidence intervals in brackets						

Adjusted for: age, gender, partnership status area of residence, and language.

*NW* North West, *NA* North America.

A logistic regression model, adjusting for age and gender, was used to examine the association between the three forms of capital (mutually adjusted) and risk of discrimination for migrants (table not shown). This showed a significant reduction in risk associated with both economic capital OR = 0.76 (CI = 0.59,0.98) and social capital OR = 0.39 (CI = 0.23,0.65), but not with cultural capital OR = 1.06 (CI = 0.90,1.25). Finally, the interaction between the capitals and perceived discrimination, did not yield significant results, nor improved the model according to the AIC/BIC criteria. We thus only report the main effects models.

## Discussion

In this study, we investigated the contribution of socioeconomic position and perceived racial discrimination to healthcare service navigation and optimisation for migrants living in Luxembourg. We found that levels of perceived discrimination varied across migrant groups, with individuals born in the Global South and in Eastern Europe having a higher prevalence of perceived racial discrimination in healthcare services compared to those born in South Europe, and Northern/Western Europe or North America. In addition, perceived racial discrimination was negatively associated with the ability to navigate and optimise healthcare services across all models. Our findings further illustrate that differences among migrant groups in terms of healthcare service navigation are explained by differences in perceived racial discrimination.

When investigating the contribution of socioeconomic position, guided by Bourdieu’s capitals, our results showed that social capital plays an important role in the effective navigation and optimisation of healthcare services. In addition, it showed that the impact of racial discrimination was reduced after accounting for social capital. Similarly, economic capital was associated with more effective navigation. The fact that the positive contribution of economic capital diminished once we adjusted for social capital indicates an important interplay between the capitals in accumulating advantage [28, 29]. While cultural capital did not make any contribution to either healthcare service navigation or optimisation in our sample, its inclusion in the models substantially increased their explanatory power. Finally, our results show that in addition to improving navigation and optimisation, Bourdieu’s economic and social capitals also limit the exposure to discrimination. Indeed, those with financial assets and effective networks might have easier access to healthcare practitioners from their own ethnic background or who show cultural competence in dealing with diverse populations. However, our hypothesis that discrimination is on the pathway leading to the devaluation of capitals, tested by including interaction terms in the models, was not confirmed in this analysis. This might be due to the lack of statistical power necessary to undertake interaction analyses or to the fact that those with higher levels of capital directly reduce their exposure to discrimination, but when exposed discrimination retains its negative impact.

The results of this study are consistent with the growing body of research suggesting that racial discrimination and different forms of capitals, both in a material and non-material form, are both equally important determinants of health outcomes [12, 30, 31]. However, it is worth recognising that the socioeconomic inequalities faced by some migrant groups reflect broader processes of racism and should not be considered to be conceptually distinct from them. This is further elaborated in Bourdieu’s notion of symbolic capital which is the recognised and legitimate form of economic, cultural and social capital [32]. By exploiting symbolic domination, the dominant group misrecognises the capitals of those being dominated as illegitimate and devalues their lifestyles, practices, physical features, knowledge, and abilities. Thus, in this context, it could be argued that the healthcare system in Luxembourg lacks the capacity to recognise the capitals of certain migrant categories as valuable. ﻿Due to structural inequalities and racism, healthcare providers may hold implicit (unconscious) and explicit biases towards certain minorities that pervade the healthcare system and negatively affect patients [33].

Although the experiences of racism among individuals from the Global South have been widely studied across United Kingdom and North America, this study adds evidence on the negative experience of individuals from the Global South and Eastern Europe in the Luxembourg healthcare context. Indeed, racial discrimination can operate on multiple levels, and capturing possible unequal treatments based on nationality, ethnic origin, religion, language and skin colour should be central to future analyses. The case of Luxembourg thus shows how racial discrimination and capital positions intersect to selectively undermine the potential benefits that should be available to everyone under a universal healthcare system. In a country in which migrants represent close to half of its residents, lines of distinction have emerged between particular migrant sub-groups that need to be considered to ensure that everyone can equally maximise the benefits from the healthcare system.

This study is the first to address migrant inequalities using a focus on navigation through and optimisation of healthcare services. It attempts to capture the experiences of diverse and hard-to-reach migrant populations who are generally underrepresented in population-based surveys [34]. The study also develops a conceptualisation of socioeconomic position, tailored to studying inequalities in healthcare settings. These findings, however, come with methodological limitations. First, while we recognise the possible endogeneity of the social capital measure, a model with a more generic social capital variable (number of close personal contacts) yielded similar results. We use self-reported measures of discrimination and of the extent to which health improved after an encounter with health professionals, which might underestimate exposure to racism and its impacts [35]. Nevertheless, there is evidence that the self-reported experience of discrimination is a valid measure of racism [36, 37]. In addition, it is worth noting that in our measure of discrimination we did group several concepts such as nationality, ethnicity, race and religion. Further research using a wider sample should attempt to capture some of the individual effects of these concepts, but also investigate how they intersect and relate to experiences of effective use of healthcare services. Second, the fact that our sample is not population-based means that our findings are not generalisable, however the approach is. This study should be seen as empirically exploring theoretical concepts able to capture the underlying mechanisms that produce social inequalities in healthcare outcomes. It also seeks to encourage further discussion on the interplay between racial discrimination and the different aspects of socioeconomic position, in shaping experiences related to healthcare.

## Conclusions

This study contributes to the growing evidence of the harmful impacts of racial discrimination, drawing on the case of a relatively egalitarian society with near universal healthcare coverage. We provide evidence that a patient’s social and racial/ethnic status may consciously or unconsciously influence the attitude of healthcare providers towards them, and thereby limit their ability to maximise the benefits of the healthcare system. For cultural, historical, and ethical reasons, data on ethnic minorities in Luxembourg, and in other mainland European countries, is scarce, limiting the comprehensive assessment of migrant health inequalities. Alongside the development of research to confirm the associations identified here, it is thus equally important for Luxembourg, and other comparable European countries, to prioritise the responsible collection of information on ethnicity and on the experience of discrimination in order to systematically track and tackle inequalities that derive from it.

Commitment to advance health equity based on socioeconomic, migration, and racial/ethnic background should be made a public health priority. Drawing on the example of the United States, it might be useful to put in place patient navigators, as well as to provide healthcare providers with cultural competence and safety training. However, structural inequalities arising from racism and socioeconomic position cannot be solved solely through individual-level interventions. Bourdieu, for example, points to the extent to which the environment in which individuals have been raised have life-long effects [28]. The impacts of racism and discrimination we have identified here signal the need for broader structural measures aimed at ensuring that all individuals are equally able, in practice as well as in policy, to appropriately access the services that maintain their health over the long-term. The interrelated mechanisms of socioeconomic position and racism need to be addressed via multilevel policies and interventions that simultaneously tackle structural, interpersonal, and institutional dimensions of racism. To achieve this, we need interventions that tackle the legal and social structures that produce socioeconomic and racial/ethnic inequalities.

## Data Availability

The datasets used during the current study is available from the corresponding author on reasonable request.

## Abbreviations

UHC: Universal Health Coverage
NW Europe & NA: North-West Europe and North America
AIC: Akaike’s Information Criteria
BIC: Bayesian Information Criteria
